# Optimizing the Reliability of Underground Utility Tunnel Localization via Multi-Source Fusion and DVAE-CNN

**DOI:** 10.3390/s26144651

**Published:** 2026-07-22

**Authors:** Shaolong Chang, Zhiguo Zhang, Xueliang Gug, Tong Zhai, Shanming Liu, Yang Zhao, Tian Guo

**Affiliations:** 1 State Key Laboratory of Information Photonics and Optical Communications, School of Electronic Engineering, Beijing University of Posts and Telecommunications, Beijing 100876, China; shaolong_chang@bupt.edu.cn (S.C.); xueliang_gu@bupt.edu.cn (X.G.); zhai_tong@bupt.edu.cn (T.Z.); 2 School of Integrated Circuit Science and Engineering, Beihang University, Beijing 100191, China; liushanming@buaa.edu.cn; 3 State Grid Beijing Electric Power Company Cable Branch, Beijing 100020, China

**Keywords:** underground utility tunnel localization, reliability, multi-source fusion, denoising variational autoencoder-CNN (DVAE-CNN)

## Abstract

This paper proposes a reliability-optimized localization method for underground utility tunnels based on multi-source fusion and a denoising variational autoencoder model. The method acquires a multi-sensor localization dataset aligned with a unified time reference. By employing a convolutional neural network-assisted denoising variational autoencoder (DVAE-CNN), it regulates localization outcomes through three key aspects: a multi-source heterogeneous data quality assessment model, a formulation of the target state transition equation, and an environmental prior-information-aided weight update strategy. This approach overcomes the low-reliability issues caused by information loss and errors in the complex environment of underground utility tunnels. Compared to localization results without reliability regulation mechanisms, the proposed method achieves an average improvement of 73.6% in localization accuracy and 85.2% in localization reliability. Finally, localization experiments conducted in an underground utility tunnel demonstrate that the proposed method can provide highly robust, reliable and continuous positioning services, indicating significant potential for application and broader adoption.

## 1. Introduction

Global Navigation Satellite Systems (GNSSs) play a crucial fundamental role in national security, social transportation and people’s daily lives. However, they fail to achieve navigation and positioning in satellite signal-shielded environments such as underground pipe galleries. Compared with outdoor open free spaces, the channel environment and spatial topology of underground pipe galleries are more complex. Radio signal transmission generally faces problems such as non-line-of-sight (NLOS) and multipath effects, which pose great challenges to the robustness of underground pipe gallery positioning methods based on wireless signal intersection. Currently, data-driven machine learning approaches are commonly adopted to overcome the difficulties generally encountered in traditional ranging-based positioning, such as NLOS, signal multipath propagation and poor stability of positioning results, thereby achieving higher-performance positioning capabilities. Most current positioning systems only consider indicators such as positioning accuracy, coverage and continuity, while rarely focusing on positioning reliability. For instance, when environmental interference, sensor anomalies and other factors lead to impaired quality of positioning data and unreliable positioning results, it often brings significant risks to subsequent location-based services (LBSs). Therefore, the reliability of positioning systems is also a key element in practical engineering applications and it is necessary to conduct in-depth research on real-time constraint and improvement methods for location reliability. With the continuous development of positioning technologies, various positioning technologies such as Bluetooth [1,2], Wi-Fi [3,4], Pseudolite positioning [5,6,7], Ultra-Wideband (UWB) [8,9], 5G/6G [10,11,12], audio [13,14], vision [15,16], geomagnetism [17,18,19] and inertial [20,21] have been continuously researched and applied worldwide. However, it is difficult for a single technology to meet the positioning requirements in complex underground pipe gallery environments, and multi-source fusion has become the mainstream technical means. Therefore, this paper focuses on systematically analyzing the research status of reliability improvement methods for current indoor positioning technology systems excluding GNSS technology. For example, Hao et al. [22] from the University of Nottingham, UK, elaborated on the important significance of reliability assessment in the field of multi-source information fusion positioning as well as related assessment methods. They noted that reliability assessment needs to comprehensively consider the measurement errors and fault characteristics of various data sources, and they concluded that the current reliability assessment methods for positioning systems generally face challenges such as complex environments, large differences in terminal states and low algorithm real-time performance. The GEOLOC Laboratory of Gustave Eiffel University in France [23] proposed a snapshot weighted least squares residual model and a sequential weighted extended Kalman filter assessment model for the multipath and NLOS issues that are mainly concerned in the reliability assessment of urban rail transit, improving the reliability of the positioning system from two aspects: accuracy enhancement and fault monitoring. Yan Wei from Wuhan University [24] proposed a reliable positioning method based on magnetic field feature matching, explored the relationship between magnetic field distribution and positioning integrity and evaluated whether the positioning system is reliable at the data level. Wang Rongxin from Xiamen University [25] proposed an integrity assessment method for vision/inertial fusion positioning systems, investigating the impact of different factors such as light intensity, camera position and target motion state on the positioning system. The team of Zhao Long from Beihang University [26] proposed a multi-mode comprehensive reliability assessment method for terrain-aided navigation systems. By establishing a sliding window uncertainty envelope to conduct consistency judgment on abnormal data and combining digital terrain to eliminate abnormal sequences, good results have been achieved.

The existing research on reliability optimization for indoor localization can be divided into three branches, with inherent limitations as follows (Table 1):

(1) Traditional variational autoencoder (VAE) and deep variational autoencoder (DVAE) only model the global distribution of 1D time series, which merely suppress single-dimensional temporal noise and fail to capture the coupling features between multi-source sensors including IMU and UWB. Current DVAE schemes are designed for general indoor scenarios, lacking a quantitative anomaly quantification mechanism for underground tunnels with severe multipath and non-line-of-sight (NLOS) interference. They only output denoised observations without quantitative data credibility indicators. (2) Convolutional neural networks (CNNs) excel at extracting local spatial features, but they have no capacity for probabilistic generation and reconstruction, so reconstruction error cannot be used to quantify observation anomalies. Existing CNN localization networks directly regress coordinates without front-end data cleaning; abnormal observations will contaminate positioning results, which cannot adapt to heterogeneous multi-source inputs. (3) Conventional particle filters adopt fixed fusion weights without geographic prior constraints from building maps, easily generating invalid cross-wall particles. Most fusion schemes only optimize positioning results in a single layer, lacking front-end anomaly elimination for raw data. Cumulative noise leads to severe trajectory drift in long narrow underground tunnels.

In summary, the existing studies only optimize localization accuracy via a single network or single stage. A two-layer trusted localization framework combining data-layer credibility evaluation and result-layer map-constrained particle filtering has not been fully investigated, which is the core research gap addressed in this paper.

## 2. Multi-Level Trusted Assessment Framework for Positioning

**Figure 1:**
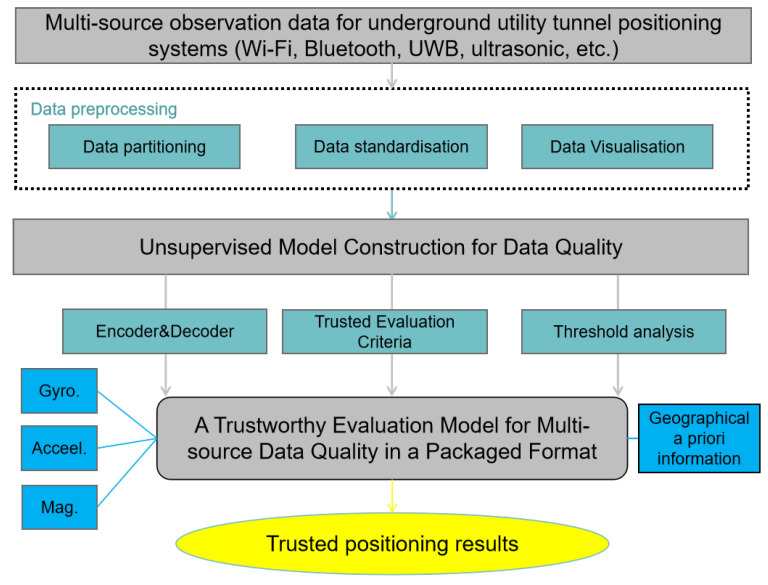
Positioning system framework with credibility constraints.

Aiming at the inherent defects of independent VAE, CNN and particle filter algorithms summarized in Section 1, this paper has designed a hybrid DVAE-CNN two-layer trusted localization framework specially for underground utility tunnel scenarios. The whole framework as illustrated in Figure 1 is divided into two collaborative stages: data-layer credibility evaluation and result-layer geographic constrained fusion positioning. In the data processing stage, the CNN enhanced denoising variational autoencoder takes multi-source heterogeneous sensor time series as input, converts one-dimensional time series into two-dimensional feature matrices, and extracts joint time-channel local features to calculate reconstruction probability, so as to quantify the credibility of each sampling data and eliminate abnormal observation values polluted by multipath and NLOS interference. On this basis, the particle filter fused with tunnel geographic prior information is adopted as the fusion positioning module. Combined with the pedestrian dead reckoning state transition equation updated by adaptive step length, the unreasonable cross-wall particle states are eliminated through tunnel boundary constraints, and dynamic weight iteration is carried out to output stable and high-reliability positioning results. This two-stage joint optimization structure makes up for the single-layer optimization limitation of existing single-model positioning methods, and it realizes the joint reliability control of raw data and final positioning trajectory. The positioning system framework comprises two core phases: offline and online. Each phase features a clear division of labor and operates collaboratively to achieve high-precision and high-credibility pedestrian positioning. In the offline phase, three core tasks are primarily accomplished: first, the collection and preprocessing of observation data from the positioning system are carried out to provide a high-quality dataset for model training; second, an unsupervised credibility evaluation model is constructed and trained and a data quality detection model meeting the real-time requirements of pedestrian positioning is obtained through lightweight design; finally, this model is packaged into an executable file to support real-time data quality evaluation in the online phase. In the online phase, based on a nonlinear fusion framework, data quality evaluation and fusion calculation of multi-source heterogeneous information are completed. Among them, the observation model innovatively integrates the positioning model and the data detection model, requiring that all data input into the fusion model must undergo credibility evaluation and analysis of data quality. This effectively filters out abnormal data and reduces its interference on the fused positioning results. In addition, in the design of the state transition model and the dynamic weight update strategy, the self-contained MEMS sensor data carried by the terminal and structured prior map information are introduced. By imposing constraints on unreasonable positioning results, the credibility and stability of the positioning results are further improved.

### 2.1. Convolution-Assisted Denoising Variational Autoencoder-Based Data Evaluation Method

**Figure 2:**
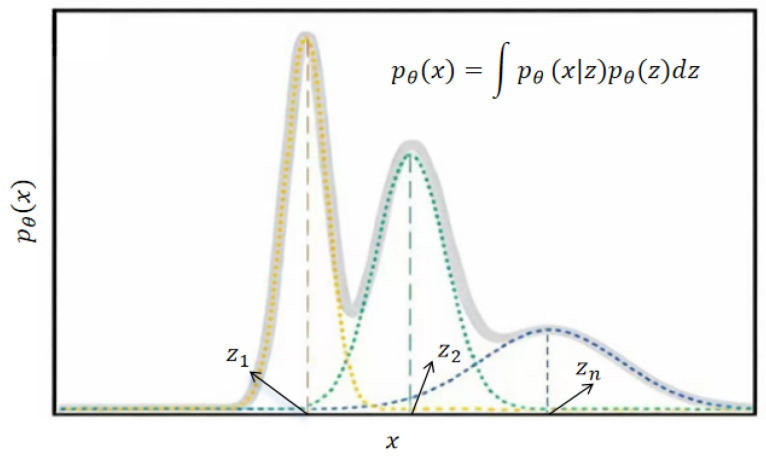
Schematic diagram of complex probability distribution composition.

This paper proposes a convolutional neural network (CNN)-assisted denoising variational autoencoder network for evaluating the quality of observation data related to positioning systems. First, the latent feature extraction part of the model is described in detail. The training objective of the model based on the variational autoencoder is to learn the parameters θ to maximize the probability density function $p_{\theta }\left(x\right)=\int p_{\theta }\left(x|z\right)p_{\theta }\left(z\right)dz$, Here, we assume that *z* follows a standard Gaussian distribution and that the prior distribution $p_{\theta }\left(x|z\right)$ follows a Gaussian distribution. Specifically, the conditional distribution of *x* given *z* satisfies:

$$x|z∼N\left(\mu \left(z\right),\sigma^{2}\left(z\right)\right)$$

where μ(z) and $\sigma^{2}\left(z\right)$ denote the mean and variance of the Gaussian distribution corresponding to *z*, respectively. In essence, p(x) is the accumulation of several distributions over the integral domain, as illustrated in Figure 2. Since *z* is closely related to the input data *x*, we can assume that $z∼p_{\theta }\left(z|x\right)$. However, in most practical problems, the posterior probability $p_{\theta }\left(z|x\right)$ is difficult to determine due to the limited amount of data. Therefore, the known distribution $q_{\phi }\left(z|x\right)$ is usually used to approximate the distribution of $p_{\theta }\left(z|x\right)$, where $q_{\phi }\left(z|x\right)$ can be determined by the VAE encoder. To make the two distributions sufficiently close, the KL divergence is commonly used to measure the similarity between the two distributions, and the parameters θ and ϕ are optimized to minimize the KL divergence, as shown in Equation (1).

(1) $$\begin{array}{cc} \mathrm{KL}\left(p(x)\;\|\;q(x)\right) & =\int_{x}p\left(x\right)\ln\frac{p(x)}{q(x)}dx\\ \; & =E_{x∼p(x)}\left[\ln\frac{p(x)}{q(x)}\right] \end{array}$$

Maximizing p(x) is equivalent to solving the maximum log-likelihood with respect to *x*, denoted as L=xlnp(x). According to the Bayesian definition:

(2) $$\begin{array}{cc} \ln p(x) & =\int_{z}q_{\phi }\left(z\right)\ln p_{\theta }\left(x\right)dz\\ \; & =\int_{z}q_{\phi }\left(z\right)\ln\frac{p_{\theta }\left(x,z\right)}{q_{\phi }\left(z\right)}dz\\ \; & \;+\int_{z}q_{\phi }\left(z\right)\ln\frac{q_{\phi }\left(z\right)}{p_{\theta }\left(z|x\right)}dz \end{array}$$

From Equations (1) and (2), we can see that the second term on the right-hand side of the equation is exactly the KL divergence between $q_{\phi }\left(z|x\right)$ and $p_{\theta }\left(z|x\right)$ and that this term is non-negative. Thus,

(3) $$\begin{array}{cc} \ln p_{\theta }\left(x\right) & \geq \int_{z}q_{\phi }\left(z|x\right)\ln\frac{p_{\theta }\left(x,z\right)}{q_{\phi }\left(z|x\right)}dz\\ \; & =\int_{z}q_{\phi }\left(z|x\right)\ln\frac{p_{\theta }\left(x|z\right)p\left(z\right)}{q_{\phi }\left(z|x\right)}dz \end{array}$$

where $\int_{z}q_{\phi }\left(z|x\right)\ln\frac{p_{\theta }\left(x|z\right)p\left(z\right)}{q_{\phi }\left(z|x\right)}dz$ is the Evidence Lower Bound (ELBO) objective function. That is, Equation (4) can be rewritten as:

(4) $$\begin{array}{cc} \ln p_{\theta }\left(x\right) & ={\mathrm{ELBO}}_{\mathrm{VAE}}\left(\theta ,\phi ,x\right)\\ \; & \;+\mathrm{KL}\;\left(q_{\phi }\left(z|x\right)\;\|\;p_{\theta }\left(z|x\right)\right) \end{array}$$

At this point, maximizing the likelihood function is transformed into maximizing the variational lower bound ${\mathrm{ELBO}}_{\mathrm{VAE}}$, which can be further decomposed as:

(5) $$\begin{array}{cc} {\mathrm{ELBO}}_{\mathrm{VAE}}\left(\theta ,\phi ,x\right) & =\int_{z}q_{\phi }\left(z|x\right)\ln\frac{p_{\theta }\left(x|z\right)p\left(z\right)}{q_{\phi }\left(z|x\right)}dz\\ \; & =-\mathrm{KL}\left(q_{\phi }\left(z|x\right)\;\|\;p_{\theta }\left(z\right)\right)\\ \; & \;+E_{q_{\phi }\left(z|x\right)}\left[\ln p_{\theta }\left(z|x\right)\right] \end{array}$$

From Equation (5), it can be seen that to maximize the variational lower bound ${\mathrm{ELBO}}_{\mathrm{VAE}}$, we usually minimize $\mathrm{KL}\left(q_{\phi }\left(z|x\right)\;\|\;p_{\theta }\left(z\right)\right)$ while maximizing $E_{q_{\phi }\left(z|x\right)}\left[\ln\left(p_{\theta }\left(z|x\right)\right)\right]$. The first term on the right-hand side of the equation represents the difference between the distribution of latent features *z* predicted from observed data *x* and the true distribution of *z*, which is also commonly interpreted as a latent space regularization term. Its purpose is to ensure that two close points in the latent space can yield similar content after decoding and that each decoded point should correspond to the input data. Therefore, it is necessary to explicitly define the regularization term. However, in real-time positioning, there are problems such as signal loss and interference in some areas, which should be taken into account during the construction and training phases of the evaluation model. This paper introduces the idea of denoising: before training, the original input data is corrupted by a corruption function to simulate noisy and interfered data; the original data is recovered through the generative model and the parameters are fine-tuned to ensure that the recovered data is close to the real data, thereby improving the anti-noise ability of the model. The corruption method adopted in this paper is the Gaussian distribution function, which maps the noisy training data to the true posterior distribution, enabling the inference network to perform more reliable training on position data. Thus, based on the variational lower bound obtained in Equation (5), we add noise to the input data *x* to obtain $\dot{x}$. At this time, the encoder becomes $q_{\phi }\left(z|\dot{x}\right)$ and the conditional Gaussian distribution satisfies $q_{\phi }\left(z|\dot{x}\right)=N\left(z|\mu_{\phi }\left(\dot{x}\right),\sigma_{\phi }\left(\dot{x}\right)\right)$, where $\mu_{\phi }\left(\dot{x}\right)$ and $\sigma_{\phi }\left(\dot{x}\right)$ are both nonlinear functions of $\dot{x}$. Assuming that the noise distribution is a known standard Gaussian distribution $p(\dot{x}|x)$ centered around *x*, we have:

(6) $$\begin{array}{cc} q_{\phi }\left(z\right) & =E_{p(x)}\left[q_{\phi }\left(z|x\right)\right]=\int_{x}q_{\phi }\left(z|x\right)p\left(x\right)dx \end{array}$$

Each time sampling $\dot{x}∼p\left(\dot{x}|x\right)$ from the distribution and substituting it into $q_{\phi }\left(z|\dot{x}\right)$ yields a different Gaussian distribution. Given that $p_{\theta }\left(\dot{x},z\right)=p_{\theta }\left(\dot{x}|z\right)p\left(z\right)$, the variational lower bound of the denoising variational autoencoder (DVAE) can be derived as:

(7) $$\begin{array}{cc} {\mathrm{ELBO}}_{\mathrm{dvae}} & =E_{q_{\phi }\left(z|\dot{x}\right)}\left[\ln\frac{p_{\theta }\left(\dot{x},z\right)}{q_{\phi }\left(z|\dot{x}\right)}\right] \end{array}$$

In Equation (7), $p_{\theta }\left(x,z\right)$ is a function of $\dot{x}$ rather than *x*. In other words, given a noisy input $\dot{x}$, the denoising variational autoencoder (DVAE) is required to recover the original data *x*, which reflects the denoising capability of the model. In the standard VAE, the process of maximizing ${\mathrm{ELBO}}_{\mathrm{dvae}}$ is actually minimizing the KL divergence between the approximate posterior probability and the true posterior probability. Thus, we have:

(8) $$\begin{array}{cc} \ln p_{\theta }\left(x\right) & ={\mathrm{ELBO}}_{\mathrm{dvae}}+E_{p_{\dot{x}|x}}\left[\mathrm{KL}\left(q_{\phi }\left(z|\dot{x}\right)\;\|\;p\left(z\right)\right)\right] \end{array}$$

Its training process consists of three steps: first, add noise to the original observed data by sampling ${\dot{x}}^{\left(m\right)}∼p\left(\dot{x}|x\right)$; second, sample latent features $z_{l}∼q_{\phi }\left(z|{\dot{x}}^{\left(m\right)}\right)$ from the encoder model; finally, reconstruct the original observed data *x* through the decoder model $p_{\theta }\left(x|z_{l}\right)$. The training process can approximate the variational lower bound of DVAE by means of Monte Carlo sampling as:

(9) $$\begin{array}{cc} {\mathrm{ELBO}}_{\mathrm{dvae}} & =E_{p(\dot{x}|x)}\left[E_{q_{\phi }\left(z|\dot{x}\right)}\left[\ln\frac{p_{\theta }\left(\dot{x},z\right)}{q_{\phi }\left(z|\dot{x}\right)}\right]\right]\\ \; & \approx \frac{1}{MK}\sum_{m=1}^{M}\sum_{k=1}^{K}\ln\frac{p_{\theta }\left({\dot{x}}^{\left(m\right)},z^{\left(k|m\right)}\right)}{q_{\phi }\left(z^{\left(k|m\right)}|{\dot{x}}^{\left(m\right)}\right)} \end{array}$$

The above content describes the basic process of feature extraction for the DVAE model, which can learn the original observed data from noisy multi-dimensional observed data and greatly improve the anti-interference ability of the model. However, the DVAE model is characterized by a favorable hierarchical distribution structure of latent variables. In the process of latent variable representation learning, the model focuses on the global features of the data but ignores the local detailed features, resulting in a certain degree of attribute preference. In fact, the observed data used for positioning is not only related to positions but also has internal correlations within the data. If local features of the data can be regarded as effective features during data feature extraction then it is possible to achieve higher-precision positioning performance. Combining DVAE with CNN in this paper helps the network perform better in terms of spatial relationships and further improves the effectiveness of feature extraction. In addition, convolutional layers are important components of deep neural networks: combining with the powerful generative model capability of autoencoders helps to reconstruct their outputs with high-fidelity encoding, thereby improving the generalization ability of the model. In the encoder, multiple convolutional layers are used for feature extraction, while in the decoder the corresponding transposed convolutional layers are employed to mirror the network structure, reconstructing the spatial structure of the data and forming a counterpart to the feature extraction in the encoding process. Generally, 1D time-series data is transformed into a 2D image-like matrix according to certain rules. By adjusting convolution kernels at different scales to extract features the model can better capture potential local correlations in the time-series data. Assuming that the input matrix size is ω, that the convolution kernel size is *K*, that the stride is *s*, and that the number of zero-padding layers is *p*, the calculation formula for the size $\omega^{\prime }$ of the feature map generated after convolution is:

(10) $$\begin{array}{c} \omega^{\prime }=\left\lfloor \frac{\omega +2p-K}{s}\right\rfloor +1 \end{array}$$

After obtaining the feature maps, a subsampling operation is performed to reduce the data volume. Similar to convolution operations, pooling also uses a sliding kernel (referred to as a sliding window). Max pooling or average pooling can be used to compress data within the sliding region, thereby reducing the complexity of the model.

Assuming a multi-channel image *V* is trained, the training process is defined as c(K,V,s) with the objective of minimizing the loss function J(V,K). Thus, during forward propagation the intermediate output *Z* is obtained via *c*, which is then passed to the rest of the network and used to compute the loss function *J*. During backward propagation, a tensor *G* is obtained that satisfies:

(11) $$\begin{array}{cc} G_{i,j,k} & =\frac{\partial }{\partial K_{i,m,n}}J\left(V,K\right)\\ \; & \;=\sum_{m,n}G_{i,m,n}V_{j,(m-1)\times s+k,(n-1)\times s+l} \end{array}$$

The gradient of *J* with respect to *V* is calculated using the following equation to propagate the error backward further:

(12) $$\begin{array}{cc} h{\left(K,G,s\right)}_{i,j,k} & =\frac{\partial }{\partial K_{i,j,k}}J\left(V,K\right)\\ \; & \;=\sum_{l,m,t}\sum_{n,p}\sum_{q}K_{q,i,m,p}*G_{q,l,n} \end{array}$$

**Figure 3:**
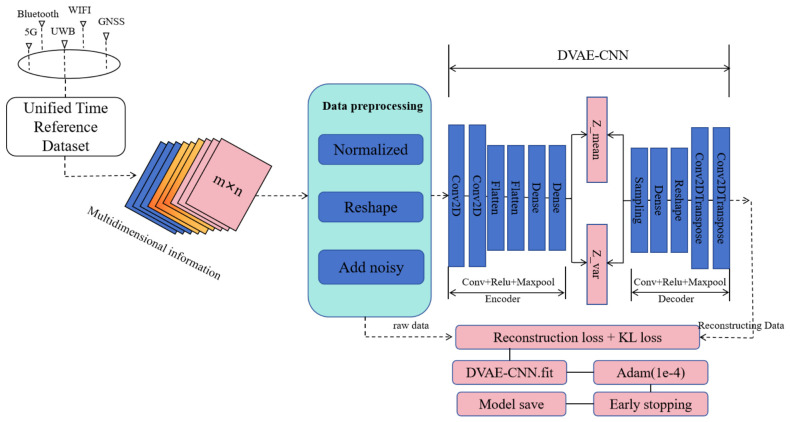
Structureand pre-training process of the DVAE-CNN model.

In the equations, *i* denotes the *i*-th output channel, *j* and *k* represent the row and column of the output, *l* is the input channel, and *m* and *n* are the row and column bias terms of the input, respectively. Generally, in the transformation from input to output nonlinear operations are implemented by adding a bias term to each channel, and this bias term can be shared across a single convolutional layer. The network framework is shown in Figure 3. For the constructed DVAE-CNN positioning model, it is necessary to first train the DVAE assisted by 2D CNN and then train the 1D CNN classifier assisted by DVAE. First, a variational autoencoder network assisted by a 2D CNN network is constructed, which is referred to as the pretraining phase. During the encoding process, a convolutional network is adopted to extract features of multi-dimensional information, and the most representative features are obtained via the max pooling method. In the decoder, transposed convolution is also used to restore the sampled latent feature vectors to obtain reconstructed images. Finally, the network is trained by optimizing the reconstruction error and KL error until the model converges.

**Figure 4:**
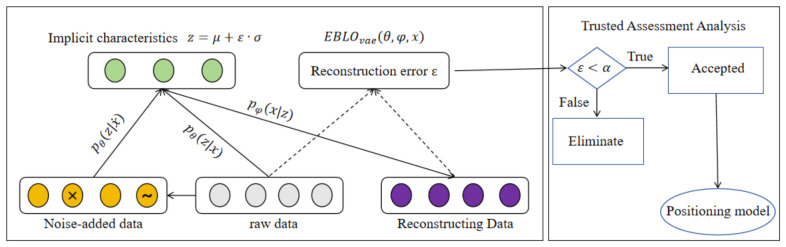
Trustworthiness evaluation model for multi-dimensional data based on reconstruction probability.

Given the strong modeling capability of the denoising variational autoencoder network for complex distributions, this section constructs a multi-source data quality evaluation framework based on reconstruction probability by combining the aforementioned model, and its schematic diagram is shown in Figure 4. The reconstruction probability from the model training in Figure 3. is used to evaluate the quality of multi-dimensional observed data, while reducing the impact of noise on anomaly detection and excluding abnormal results from being introduced into the fusion framework as observed values, thereby improving the reliability and accuracy of the system. Generally, after the completion of model training the reconstruction probability of normal data is low while that of abnormal data is high. Therefore, abnormal data is identified by setting a reconstruction probability threshold, which effectively reduces the impact of abnormal data and improves positioning accuracy. As can be seen from the following equation, the reconstruction probability can be expressed as:

(13) $$\begin{array}{cc} E_{z∼p_{\theta }\left(z|x\right)}\left[\ln q_{\phi }\left(x|z\right)\right] & =\frac{1}{L}\sum_{l=1}^{L}\ln q_{\phi }\left(x|z_{l}\right) \end{array}$$

where *L* denotes the number of samples drawn from the distribution $q_{\phi }\left(x|z\right)$. In Figure 4, α is the set evaluation threshold, which can be determined through experiments by considering factors such as positioning performance and environmental conditions. The training process is presented in Algorithm 1 as follows.
**Algorithm 1** Training and Application of Multi-Source Data Credibility Evaluation Model**Require:** Multi-source data *X*; credible evaluation threshold α**Ensure:** Evaluation model *B*  1:**Data Preprocessing**: Normalize data, intercept input data, add noise, divide data into training/test/validation sets  2:**while** encoder model training not completed **do**  3:    Initialize DVAE-CNN model and set hyperparameters  4:    Load training dataset  5:    Obtain mean, variance and latent features *z* of samples  6:    Get reconstructed data via decoder  7:    Calculate reconstruction error *e* by comparing reconstructed data with original data  8:    **if** *e* converges to the set threshold **then**  9:        Trigger early stopping mechanism to end training10:    **else**11:        Go to Step 612:    **end if**13:    Fine-tune the network and update parameters14:    Repeat Steps 4–6 until model converges15:**end while**16:Save model parameters17:Evaluate the map x(t) at time *t* using η18:**while** New measurements are being performed **do**19:    **if** Reconstruction error e<α **then**20:        Input the data into the positioning model21:    **else**22:        Eliminate the data at current time23:        Repeat Step 1224:    **end if**25:**end while**

### 2.2. Credibility Regulation Method for Indoor Positioning Based on Multi-Source Heterogeneous Information

**Figure 5:**
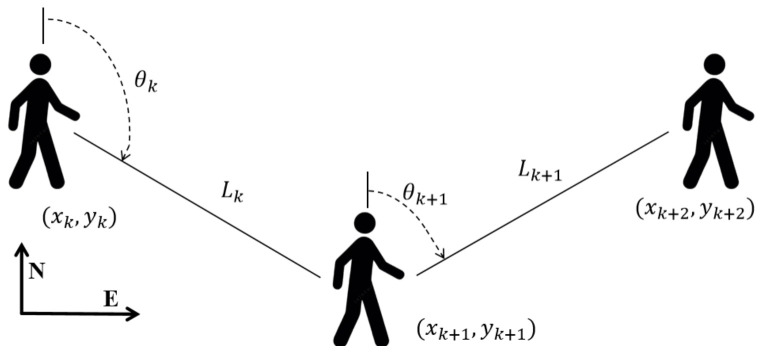
PDR algorithm schematic illustration.

In the previous section, credibility constraints for the indoor positioning system were implemented at the data level. Actually, the performance of indoor positioning is closely related to the surrounding geographical environment. Similar to indoor building maps that contain abundant prior information, combining positioning results with indoor map information can reduce the uncertainty of positioning results; matching map information with sensor data (such as Wi-Fi signals, Bluetooth beacons, inertial measurement units (IMUs), etc.) is conducted to optimize positioning results; the combination of indoor maps and ray tracing methods can improve the measurement accuracy of radio signals and correct the cumulative error of inertial positioning based on indoor scene perception. Therefore, exploring the prior information of indoor building structures to constrain positioning results will be an effective method for improving credibility. In addition, most current user terminals (such as smartphones) are equipped with various types of inertial devices, which can provide continuous time-series information and can also be used as a regulatory means for credible positioning. For example, MEMS sensor information combined with established criteria is used to eliminate results that do not conform to pedestrian movement laws, thereby reducing the uncertainty of positioning results. In summary, this paper introduces a nonlinear particle filter algorithm as a “position filter” for building map information and pedestrian movement information. Through corridor boundaries, doors, windows and other building structures, the position distribution of the particle swarm is restricted. The specific process includes the following five steps:

(1) In the initialization stage, it consists of two parts: particle state space initialization and positioning model initialization. The particle set is defined as $H=\{x_{i}|i=1,2,\ldots ,n\}$, where *n* is the number of particles and the particle state space includes position coordinates and initial movement step size $\left(x,y,L_{0}\right)$. The initialization of the positioning model generally involves constructing the same network model as that used in training before positioning execution, loading model parameters and interfaces to receive data in real time for position estimation.
(2) In the target position prediction stage, the pedestrian dead reckoning (PDR) algorithm is implemented based on terminal MEMS sensors, as shown in Figure 5. Position prediction is completed via the following state transition equation for particles:
  (14) $$\left[\begin{array}{c} x_{k}\\ y_{k} \end{array}\right]=\left[\begin{array}{c} x_{k-1}\\ y_{k-1} \end{array}\right]+\left[\begin{array}{c} L_{k}\cdot \sin\theta \\ L_{k}\cdot \cos\theta \end{array}\right]$$
  where the position at time k−1 is $\left(x_{k-1},y_{k-1}\right)$, the position coordinates at time *k* are $x_{k}$, and $y_{k}$ and θ is the moving direction of the particles. Due to the low detection accuracy of direction sensors, θ is set as a random number in this paper to ensure the diversity of the particles.

**Figure 6:**
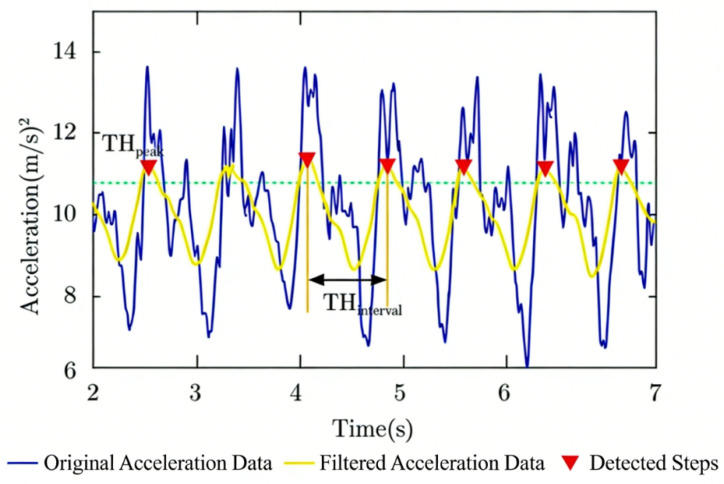
Variation characteristics of acceleration information during pedestrian movement.

Since the step length of pedestrians is random, it is affected by factors such as height, gender and walking speed for different individuals. Therefore, if the particle moving step length or speed is set to a fixed value in the state transition equation then certain errors will be introduced, making the positioning results unreliable (usually manifested as lag or offset of positioning results). To address this issue, this paper combines MEMS sensor information to dynamically estimate pedestrian motion states and adaptively update the particle moving step length, thereby improving the reliability of the system. Specifically, the acceleration information of pedestrians exhibits periodic changes during normal walking, and the number of walking steps can be identified through such changes. To make the detection method independent of terminal posture, the magnitude of three-axis acceleration $a_{\mathrm{mer}}=\sqrt{a_{x}^{2}+a_{y}^{2}+a_{z}^{2}}$ is generally used as the basis for step detection. At this time, step detection is completed by setting the peak detection threshold and the peak-valley spacing threshold. In Figure 6, the blue curve represents the original acceleration data, the yellow curve denotes the data after low-pass filtering, and the red triangles indicate the detected number of steps. Similarly, step length estimation can be completed based on acceleration information and step detection results. Relevant studies have proposed numerous step length estimation models, including linear models, nonlinear models and deep learning models. In this paper, the step length estimation model shown in the following equation is used to dynamically update the state transition equation:

(15) $$\begin{array}{cc} L & =\alpha \times 4\sqrt{a_{\max}-a_{\min}}+\beta \times f_{s}+\gamma \times v_{s}+\delta \times w_{s} \end{array}$$

where $a_{\max}$ and $a_{\min}$ correspond to the maximum and minimum values of acceleration during a single-step process, respectively; α, β, γ and δ represent the step length parameters determined under different human activity states; $f_{s}$, $v_{s}$ and $w_{s}$ denote the activity frequency of pedestrians, the standard deviation and the variance of acceleration, respectively. Through the above steps, adaptive particle state update is realized, which further improves the degrees of freedom of system positioning.

**Figure 7:**
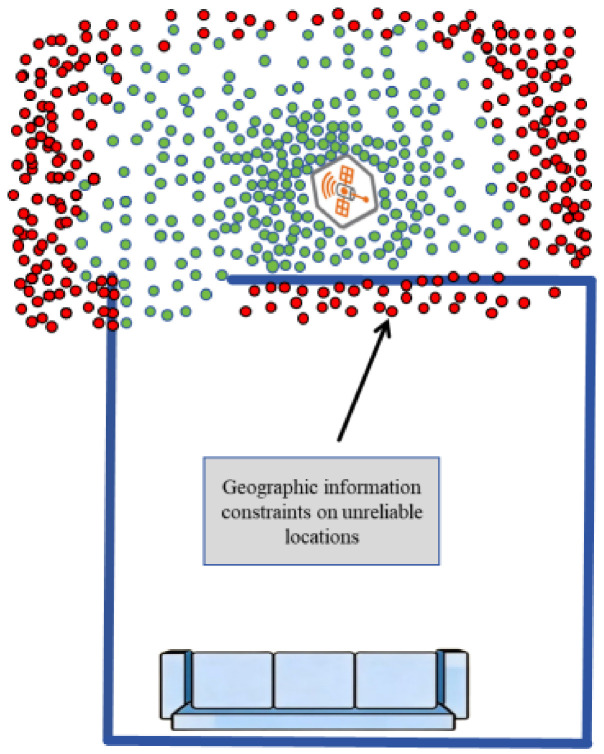
Geographical prior information-assisted position credibility constraints.

(3) In the weight update stage, weights are updated by comparing the predicted measurement values with the probability distribution function obtained from the actual measurement process. In practical applications, since some particles may exhibit unreasonable situations such as “wall-penetration” during the state update process, real environmental information is considered as the basis for credible constraints in the weight update stage, as shown in Figure 7. In Figure 7, green dots represent the possible positions where the user may move in the next moment and red dots denote the untrustworthy regions of particle swarm distribution.
(4) In the resampling process, each particle is assigned a corresponding weight. Particles with low weights are discarded since they deviate greatly from the actual user state. Thereafter, all particles are aggregated around high-weight regions to facilitate the convergence of the particle swarm. The effective particle number threshold is defined as $N_{\mathrm{eff}}=N/2$, and resampling is triggered once the actual effective particle number is lower than this threshold according to the particle weights. Comparative experiments were carried out with different particle quantities (100, 200, 300, 400) and various resampling thresholds. Our experimental results indicate that the variation range of the average positioning error is less than 0.06 m when the particle number ranges from 200 to 400. Moreover, the system achieves stable operation when the resampling threshold is selected within the interval from 0.4N to 0.6N. Consequently, the particle number is finally set to 200 to realize a favorable trade-off among positioning accuracy, real-time performance and computational overhead.
(5) In the position estimation stage, the weighted average value of all the particles is taken as the estimated position at the current moment.

In summary, the designed real-time position credibility evaluation method mainly consists of a multi-dimensional data credibility evaluation model and prior information constrained measurement: the credibility of the positioning results is improved through the joint constraints of the two, and the algorithm flow is presented in Algorithm 2, as follows.
**Algorithm 2** Methods for Enhancing Position Reliability**Require:** Prior map information, number of particles *n*, particle step length *L*, credible evaluation threshold α, resampling threshold τ**Ensure:** Reliable positioning result *P*  1:**Initialization**: Sample a set of particles from the initial state distribution  2:**while** positioning result is not obtained **do**  3:    **for** each particle **do**  4:        Update current position via Equation (14)  5:        Update weight information using:$\omega =\frac{1}{\sqrt{2\pi }\sigma_{\omega }}\exp\left\{-\frac{{\left|s-p\right|}^{2}}{2\sigma_{\omega }^{2}}\right\}$
        where *s* is the particle state at current moment and $\sigma_{\omega }$ is the measurement deviation  6:        **if** particle penetrates walls (or building structures) **then**  7:            Set the weight of the corresponding particle to 0 (eliminate potential abnormal positions)  8:        **end if**  9:        **if** number of particles <τ **then**10:            **Resampling**: Generate new particles based on weights (polynomial resampling)11:        **end if**12:        Obtain current positioning result via particle states and weights13:    **end for**14:**end while**15:Output reliable positioning result *P*

## 3. Experimental Verification and Application

This section mainly includes two sets of experiments. The first set verified the effectiveness of the proposed credibility evaluation model and the second set introduced the practical application effect of the results of this study in positioning within underground utility tunnels. Both the experiments were run on an industrial laptop with Intel i7 CPU and 16 GB RAM. The algorithm was implemented in Python 3.9 with PyTorch for model training, while the raw sensing data were processed by MATLAB R2023b. The test hardware included DW1000 UWB modules, wearable IMUs and a Leica total station for ground truth calibration.

### 3.1. Dataset and Test Platform

For this paper, we constructed two groups of datasets collected from simulated laboratory tunnel and real underground utility tunnel, with the full detailed parameters summarized in Table 2.

### 3.2. Effectiveness Test of the Proposed Credibility Evaluation Method in Experimental Environment

**Figure 8:**
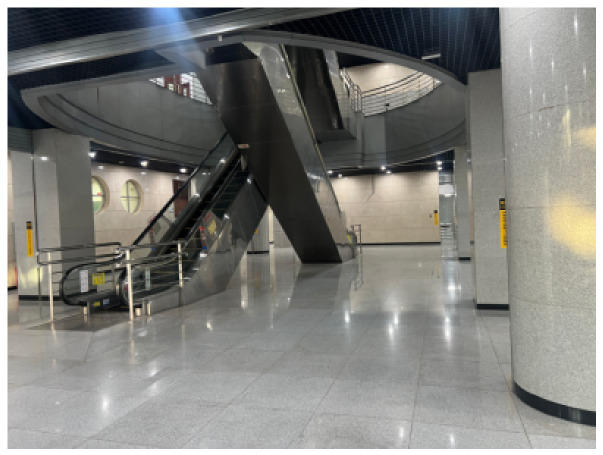
Photographs of the real experimental environment.

**Figure 9:**
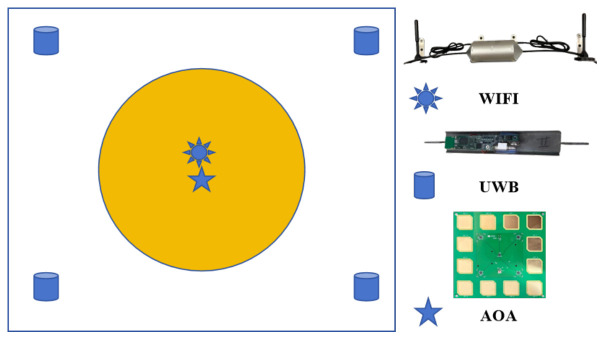
Schematic layout of multi-source positioning anchor nodes (UWB, Wi-Fi, AOA).

To verify the effectiveness of the credibility evaluation framework, for this section we conducted tests in an indoor laboratory environment with a test area of approximately 18 m × 24 m; the test environment map is shown in Figure 8. The existing positioning test network deployed in this environment integrates UWB, Bluetooth AOA and Wi-Fi modules. The benchmark positioning scheme adopted in this experiment is denoted as the reference positioning system in the following text. A total station was utilized to calibrate the ground-truth coordinates of all the test points, which served as the benchmark for evaluating the localization accuracy. A high-precision total station was adopted to acquire ground-truth positions. Its horizontal positioning accuracy was ±(1mm+2ppm·D), where *D* denotes the test distance. The measurement error was less than 3 mm within the entire test range. This guaranteed the accuracy and reliability of the positioning error calculation. The proposed system integrates three mutually complementary positioning modules. The Wi-Fi positioning module achieves a coverage distance of 100 m with a positioning accuracy of 3 m. The ultra-wideband (UWB) positioning module supports 100 m coverage and provides positioning accuracy up to 1 m. The Bluetooth angle-of-arrival (AOA) positioning module has an effective coverage of 50 m and a positioning accuracy of 0.8 m. As shown in Figure 9, the Wi-Fi and Bluetooth AOA devices were mounted overhead at the central area of the experimental site, whereas the UWB nodes were deployed at the four corners of the test area.

**Figure 10:**
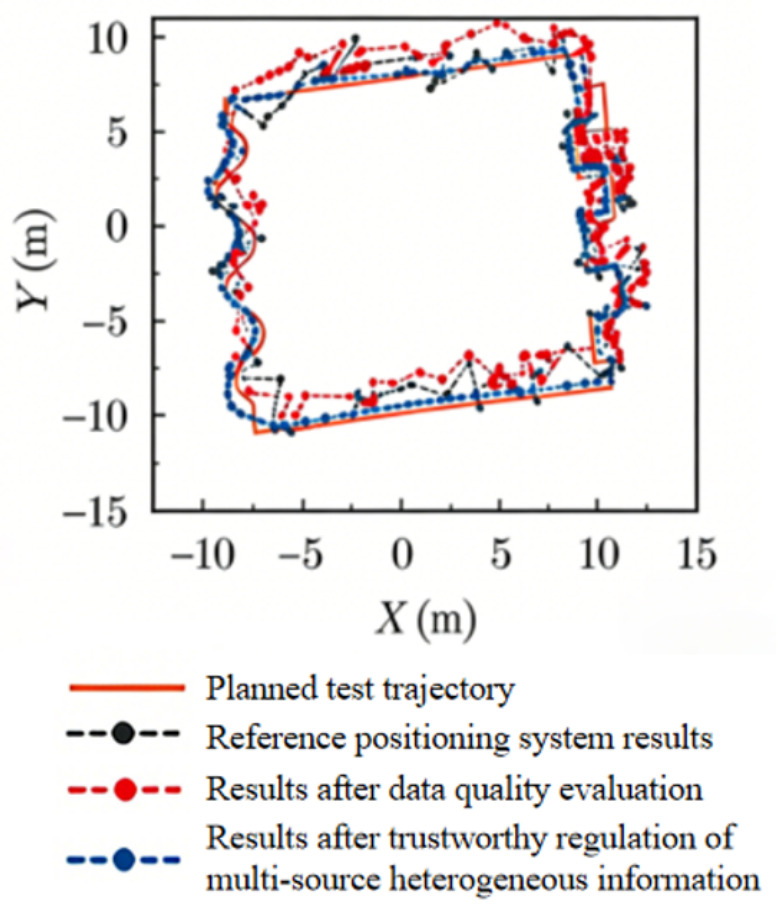
Effectiveness analysis results of the credibility evaluation mechanism.

For this paper, Keras, a commonly used deep learning library, was selected as the construction tool for the network model. The construction process consists of five parts: model selection, network construction, compilation, training and prediction. In network construction, a denoising variational autoencoder network including an input layer, convolutional layers, pooling layers and fully connected layers is designed. Among these, feature extraction is realized through multi-layer convolutional operations. The backpropagation algorithm is used to train the entire network, enabling the trained model to find the nonlinear mapping relationship between the reference class and the position information. Specifically, when the loss function between adjacent iterations drops below the threshold or the number of iterations is satisfied, the network reaches stability and the network parameters are saved. To save time and resources, for this paper we adopted the early stopping strategy for training, and we repeatedly trained using cross-validation until the model converged. Our tests were conducted in the environment shown in Figure 8, and a comparative analysis was performed on the effectiveness of the multi-source data quality evaluation model and the multi-source heterogeneous information-assisted position constraint method. The test results are shown in Figure 10.

**Figure 11:**
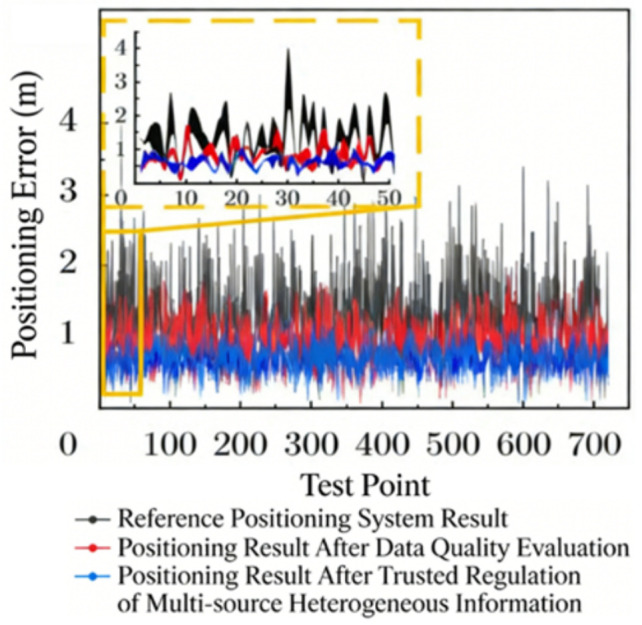
Positioning error band diagram.

**Figure 12:**
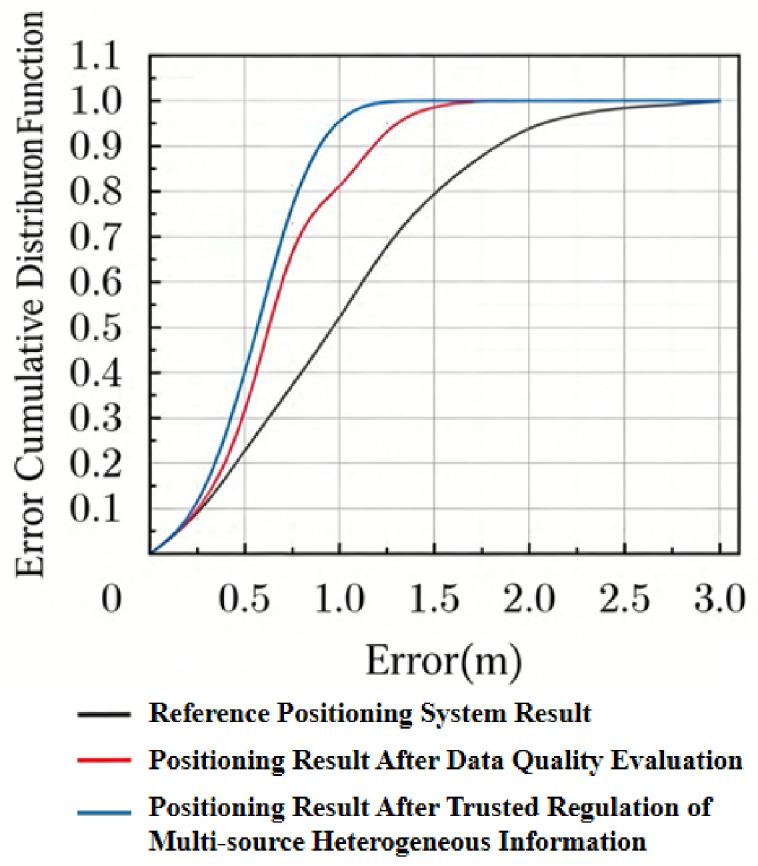
Positioning error analysis.

The test results are shown in Figure 10, where the black trajectory is the trajectory of the reference positioning system and the red trajectory is the positioning result after adding the observation data quality evaluation model. Real-time data quality analysis and abnormal data elimination made the positioning trajectory smoother. On this basis, prior map and MEMS sensor data were introduced as references for new particle weight assignment to further regulate the positioning results and achieve more smooth and credible outcomes. In summary, after introducing the credibility regulation mechanism the positioning accuracy and credibility significantly improved. To analyze the effectiveness of the credibility evaluation mechanism more clearly, the error band diagram and error cumulative distribution function of the trajectory were plotted, as shown in Figure 11 and Figure 12. The error band was selected instead of the error curve to evaluate the positioning error through the confidence interval more scientifically and objectively. The blue error band in the figure is the result after the credibility evaluation framework, with an average positioning error of 0.51 m, a maximum positioning error of 1.31 m, and 94% of the errors less than 1 m. Compared with the positioning results without the credibility evaluation, the average positioning accuracy improved by 73.6%. In addition, the reliability of a positioning system is usually defined as the percentage of the total observation time that the system’s positioning accuracy is less than the reliability threshold within a specified area and period. When the reliability threshold was set to 1 m in this paper, the positioning reliability after credibility evaluation improved by 85.2%, which further verified the effectiveness of the evaluation system.

### 3.3. Effectiveness Test of the Credibility Evaluation Method in the Underground Utility Tunnel Environment

**Figure 13:**
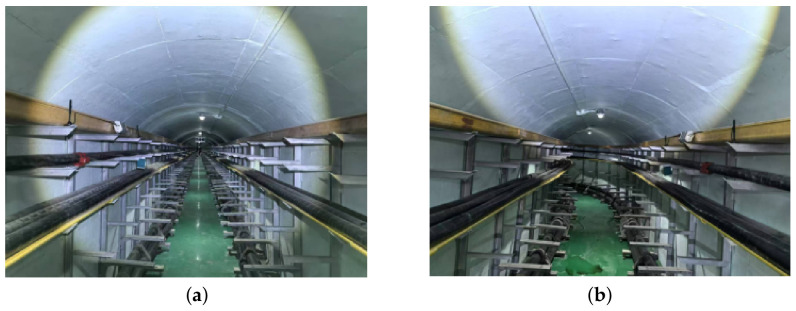
Internal structure diagram of underground utility tunnel. (**a**). Small-scale spaces with severe metallic interference. (**b**). Spatial twist switching.

To evaluate the practical performance of the proposed trust evaluation method, field tests were conducted in a complex underground utility tunnel in Beijing, as illustrated in Figure 13. The tunnel environment imposed severe challenges for field deployment due to the lack of Wi-Fi coverage and available power supply. To tackle these issues, a self-developed Wi-Fi system based on fiber-enabled simultaneous power and signal transmission was deployed to provide continuous network coverage and energy supply for the tunnel scenario. Furthermore, multiple positioning technologies, including ultra-wideband (UWB), Bluetooth, and inertial measurement unit (IMU) sensing, were fused to achieve reliable positioning in the complex underground tunnel environment.

**Figure 14:**
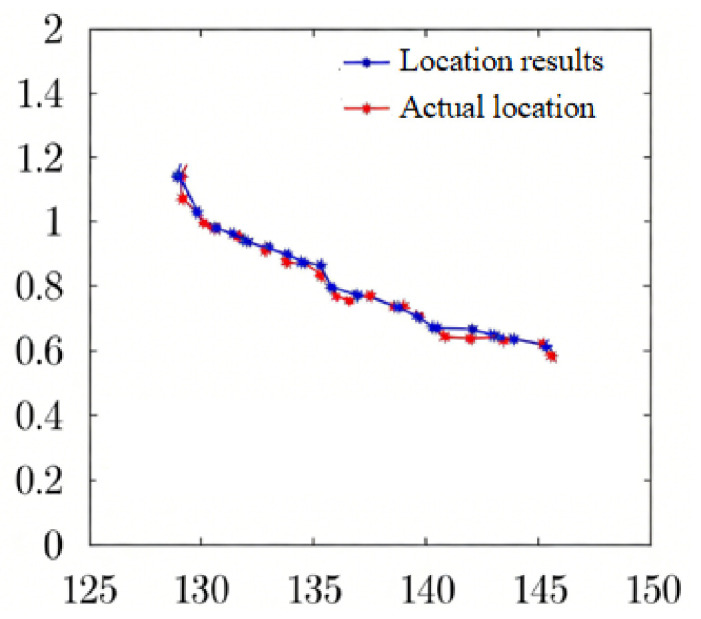
Comparison of positioning results and actual locations.

The tester moved through the underground utility tunnel while holding the test equipment, and a total station was used to mark the actual positions along the trajectory for positioning accuracy evaluation. The test results are shown in Figure 14. In the specific environment of the underground utility tunnel, and with the assistance of geographical prior information-based position credibility constraints, the Y-axis error was extremely small and the main error was concentrated on the X-axis. The maximum error did not exceed 0.87 m and the average error was 0.46 m, which enabled high-precision continuous positioning coverage of the monitoring area.

### 3.4. Applicable Scope and Failure Conditions of the Proposed Method

This subsection clarifies the applicable scenarios, engineering deployment requirements and failure boundary conditions of the designed DVAE-CNN two-layer trusted localization framework, to facilitate subsequent academic research and practical underground deployment.

#### 3.4.1. Applicable Scenarios and Deployment Prerequisites

The proposed method is mainly oriented to enclosed narrow underground spaces with structured wall geographic prior maps, such as municipal utility tunnels, comprehensive pipe corridors and pedestrian underground passages. The framework takes MEMS inertial measurement data and UWB ranging observation values as multi-source heterogeneous input, and it obtains the optimal positioning reliability under moderate multipath/NLOS interference and normal low-speed pedestrian walking states.

For on-site engineering deployment, two necessary preconditions should be satisfied:

1. A rasterized vector structural map of the underground scene, which provides wall boundary constraints for particle filter trajectory correction;
2. UWB anchor nodes deployed at intervals of 10–15 m inside the corridor, matching low-cost wearable IMU terminals for pedestrian data collection.

#### 3.4.2. Failure Boundary and Performance Degradation Mechanism

The algorithm will produce severe positioning drift or complete failure under four extreme invalid conditions, and the corresponding internal degradation reasons are analyzed as follows:

1. Long-time full-band signal occlusion with UWB observation loss rate higher than 70%. Massive missing ranging data cannot support valid credibility evaluation via DVAE-CNN, resulting in unfiltered abnormal observations entering the fusion module.
2. Unstructured open underground spaces without available wall map constraints. The particle filter loses geographic boundary correction, and cumulative inertial drift cannot be suppressed.
3. Extreme high-speed running or frequent sharp turning motions. The periodic acceleration feature for step length estimation is destroyed, leading to completely invalid adaptive step updating.
4. Sustained dense NLOS interference, where the reconstruction error continuously exceeds the pre-set threshold α. All observed data are marked as invalid, and the fusion positioning module lacks effective measurement inputs.

## 4. Conclusions

By sufficient ablation experiments, threshold sensitivity analysis and field tests on real underground tunnels, jointly, we have verified that the proposed framework achieves prominent improvements in positioning accuracy and reliability compared with pure VAE, CNN and traditional particle filter baselines. To address the low credibility of positioning systems in complex underground utility tunnel environments, this paper proposes a multilevel credibility enhancement method that jointly optimizes the positioning data layer and result layer. First, a hybrid DVAE-CNN network model is constructed to establish the mapping relationship between positioning data credibility and latent features. This strategy effectively mitigates environmental interference on raw observation data and improves positioning credibility at the data source. Second, a nonlinear filtering framework is developed to integrate the multi-source heterogeneous data quality evaluation model, geographic prior information, and MEMS sensor data. By optimizing state transition constraints and dynamic weight updating strategies, the credibility of the final positioning results is further improved. In comparison, conventional positioning methods suffer from severe performance degradation under non-line-of-sight (NLOS) conditions, multipath effects, and strong electromagnetic interference. Standard VAE and CNN models lack reliable adaptive adjustment mechanisms, leading to unstable positioning performance in time-varying underground scenarios. Furthermore, traditional particle filter algorithms ignore data quality evaluation procedures, resulting in significant error fluctuations and insufficient system robustness. Benefiting from the embedded data quality assessment, adaptive state transition mechanism, and geographic prior constraints, the proposed method can effectively suppress abnormal positioning errors and enhance overall system robustness. Our experimental results demonstrated that the proposed method achieved an average positioning error of 0.51 m and a maximum error of 1.31 m, with 94% of positioning errors below 1 m. Compared with the baseline methods without credibility evaluation, the proposed method improved average positioning accuracy and positioning reliability by 73.6% and 85.2%, respectively. The proposed method has been successfully deployed in practical underground tunnel projects in Beijing to support high-reliability continuous positioning. It provides solid theoretical and technical support for the large-scale promotion and practical application of high-credibility positioning technology in underground utility tunnel scenarios.

## Figures and Tables

**Table 1:** Comparison between the proposed framework and existing mainstream localization methods.

Method	Cred.	Cross-Sen. Feat.	Geo. Const.	Two-Stage	Tunnel Adapt.
VAE/DVAE	No	Unsupported	None	Single denoise	Poor
CNN Loc.	No prob.	Spatial only	None	Single reg.	Poor
PF	No pre-filter	None	Weak	Single fusion	Poor
Ours DVAE-CNN PF	Rec. prob.	2D joint feature	Hard wall	Dual-layer	Optimized

**Table 2:** Detailed configuration of the collected positioning dataset.

Item	Details
Test scenarios	18 m × 24 m laboratory; 1260 m real power underground utility tunnel
Volunteers	Eight participants (four male, four female), height range: 160–185 cm
Total trajectories	42 lab trajectories + 36 field trajectories = 78 full trajectories
Single trajectory duration	60–300 s, total raw data collection time: 12.8 h
Sensor hardware	Nine-axis MEMS IMU (accel. ±16 g, gyro. $\pm 2000^{\circ }$/s); UWB anchors and tags
Sampling frequency	IMU: 100 Hz; UWB ranging: 10 Hz
Train/val/test split	70% training, 15% validation, 15% test, split by complete trajectories to avoid data leakage
Environmental interference	Metal multipath reflection, weak magnetic field, pedestrian occlusion, random pulse noise
Data release statement	Both the laboratory simulated data and the field tunnel measurement data involved internal engineering confidential information of the project, and the dataset will not be publicly released. Relevant experimental reproduction guidance can be provided to reviewers for manuscript evaluation only.

## Data Availability

The raw data supporting the conclusions of this article will be made available by the authors on request.
